# Molecular Insights into Binding Mode and Interactions of Structure-Based Virtually Screened Inhibitors for *Pseudomonas aeruginosa* Multiple Virulence Factor Regulator (MvfR)

**DOI:** 10.3390/molecules26226811

**Published:** 2021-11-11

**Authors:** Raed A. H. Almihyawi, Halah M. H. Al-Hasani, Tabarak Sabah Jassim, Ziyad Tariq Muhseen, Sitong Zhang, Guang Chen

**Affiliations:** 1 College of Life Sciences, Jilin Agricultural University, Changchun 130118, China; raed.alamery@yahoo.com; 2 Department of Quality Control, Baghdad Water Authority, Baghdad 10011, Iraq; 3 Department of Biotechnology, College of Science, University of Diyala, Baqubah 32001, Iraq; halahalhasani@sciences.uodiyala.edu.iq; 4 Department of Prosthodontic Technologies, Dijlah University College, Baghdad 00964, Iraq; tabarak.sabah@duc.edu.iq; 5 School of Life Sciences, Shaanxi Normal University, Xi’an 710119, China; ziyad.tariq82@gmail.com; 6 Key Laboratory of Ministry of Education for Medicinal Plant Resource and Natural Pharmaceutical Chemistry, Shaanxi Normal University, Xi’an 710119, China; 7 Key Laboratory of Straw Biology and Utilization, Ministry of Education, Jilin 130118, China

**Keywords:** *Pseudomonas aeruginosa*, multiple virulence factor regulator, asinex antibacterial library, comprehensive marine natural products database, M64 control, binding free energies

## Abstract

Multi-drug resistance (MDR) bacterial pathogens pose a threat to global health and warrant the discovery of new therapeutic molecules, particularly those that can neutralize their virulence and stop the evolution of new resistant mechanisms. The superbug nosocomial pathogen, *Pseudomonas aeruginosa*, uses a multiple virulence factor regulator (MvfR) to regulate the expression of multiple virulence proteins during acute and persistent infections. The present study targeted MvfR with the intention of designing novel anti-virulent compounds, which will function in two ways: first, they will block the virulence and pathogenesis *P. aeruginosa* by disrupting the quorum-sensing network of the bacteria, and second, they will stop the evolution of new resistant mechanisms. A structure-based virtual screening (SBVS) method was used to screen druglike compounds from the Asinex antibacterial library (~5968 molecules) and the comprehensive marine natural products database (CMNPD) (~32 thousand compounds), against the ligand-binding domain (LBD) of MvfR, to identify molecules that show high binding potential for the relevant pocket. In this way, two compounds were identified: Top-1 (4-((carbamoyloxy)methyl)-10,10-dihydroxy-2,6-diiminiodecahydropyrrolo[1,2-c]purin-9-yl sulfate) and Top-2 (10,10-dihydroxy-2,6-diiminio-4-(((sulfonatocarbamoyl)oxy)methyl)decahydropyrrolo[1,2-c]purin-9-yl sulfate), in contrast to the co-crystallized M64 control. Both of the screened leads were found to show deep pocket binding and interactions with several key residues through a network of hydrophobic and hydrophilic interactions. The docking results were validated by a long run of 200 ns of molecular dynamics simulation and MM-PB/GBSA binding free energies. All of these analyses confirmed the presence of strong complex formation and rigorous intermolecular interactions. An additional analysis of normal mode entropy and a WaterSwap assay were also performed to complement the aforementioned studies. Lastly, the compounds were found to show an acceptable range of pharmacokinetic properties, making both compounds potential candidates for further experimental studies to decipher their real biological potency.

## 1. Introduction

Infectious diseases are a major reason for human disorders, particularly in low income countries [1,2]. Infectious diseases have been the top cause of deaths around the globe for a long time and have high economic costs [3,4]. Multi-drug-resistant bacterial species emerged as a serious threat to public health and are classified by the World Health Organization (WHO) as one of the top 10 health problems that humanity is currently facing [5,6,7]. Antibiotic resistance, in particular, is of great concern in six highly virulent bacterial species (*Enterococcus faecium*, *Staphylococcus aureus*, *Klebsiella pneumoniae*, *Acinetobacter baumanii*, *Pseudomonas aeruginosa*, and *Enterobacter* spp.) (commonly referred to as ESKAPE pathogens) [6,8]. The design of new drugs against the mentioned antibiotic-resistant bacterial pathogens involves a constant search and the unveiling of new chemically diverse molecules to tackle ESKAPE pathogens requires more time [9].

Gram-negative bacilli of the genus *Pseudomonas* are found in freshwater, soil, and marine environments [10]. *P. aeruginosa* is a frequent causative pathogen of nosocomial infections such as bacteremia, pneumonia, urinary tract infections, meningitis, and damaged mucous membranes or skin, the latter enabling pathogens to enter the blood circulation and cause septicemia [11]. The infectious bacteremia caused by *P. aeruginosa* has a higher mortality rate than other species of *Pseudomonas* due to its higher resistance spectrum against many of the antibiotics [12]. It is a ubiquitous pathogen that has the natural ability to thrive in moist environments and show resistance to many antiseptics and antibiotics, and thus, is commonly found in hospital intensive care units [13]. The resistance is multifactorial and is mediated by porins, penicillin-binding proteins, efflux pumps, chromosomal β-lactamases, and aminoglycoside-modifying enzymes, all of which contribute to resistance against antibiotics that are commonly used for treating *P. aeruginosa* infections [14]. The multi-drug resistance in this pathogen has made it critical to come up with new antimicrobial drugs. 

*P. aeruginosa* survives the action of antibiotics through the formation of dormant cells known as antibiotic-tolerant/persister (AT/P) cells [15]. In these cells, the metabolic state is suppressed, enabling tolerance to lethal antibiotic concentrations. It was demonstrated that multiple virulence factor regulator (MvfR) plays a key role in the formation of AT/P cells and the regulation of different virulence functions in *P. aeruginosa* [16]. In order to block the function and to design anti-virulent drugs, the current study uses different applications of computer aided drug design (CAAD) [17]. Computational approaches are of significant importance in the process of drug discovery and development [18,19,20]. The search for specific and selective novel drug targets against bacterial pathogens is an important step in the design of new drug molecules to fight bacterial infections. This in silico study aims to identify potential inhibitory molecules against *P. aeruginosa* that can be developed as drugs. The objective is to screen high-affinity binders from antibacterial and natural databases. Virtual screening was performed to prioritize the best-docked molecule for the MvfR, followed by a biophysical analysis of molecular dynamics simulation and binding free energies to validate the docking predictions. The findings of this study can help in the identification of novel leads against nosocomial *P. aeruginosa* infections. 

## 2. Materials and Methodology

### 2.1. Retrieval of MvfR and Preparation

Initially, the crystal structure of *P. aeruginosa* MvfR was retrieved from the protein data bank (PDB) using the PDB ID of 6B8A [21]. The MvfR crystal structure was of 2.65 Å resolution, and had an R-Value Free score of 0.251 and an R-Value Work score of 0.216 [16]. The enzyme was visualized in UCSF Chimera version 1.15 [22], and was analyzed to prepare it for the molecular docking study. The water molecules and associated co-crystallized ligand (M64 compound) were deleted from the protein structure. The structure then entered the energy minimization phase of 2000 steps: 1000 steps of the steepest descent algorithm (to ease highly unfavorable clashes) and 1000 steps of the conjugate gradient algorithm (a slower algorithm that is effective at reading the energy minimum). The said algorithms were run at a default step size of 0.02 Å. AMBER ff14SB [23,24] was used to assign charges to the protein residues. 

### 2.2. Ligands Library Preparation 

In order to discover novel chemical entities of good safety and strong antibacterial activity, valuable starting leads are indeed of significance. To obtain these, several antibacterial libraries were utilized for use in structure-based virtual screening. The libraries employed in this study included the Asinex antibacterial library (~5968 molecules) (http://www.asinex.com/?page_id=14/, accessed on 7 August 2021) and the comprehensive marine natural products database (CMNPD) (~32 thousand compounds) [25]. Both libraries were imported to FAF*Drugs*4 (https://mobyle.rpbs.univ-paris-diderot.fr/cgi-bin/portal.py?form=FAF-Drugs3#forms::FAF-Drugs4, accessed on 8 August 2021) [26] to be filtered based on Lipinski’s rule of five [27,28]. The filtered compounds were then transferred to PyRx v0.8 [29] and converted into .pdbqt, and their energy was minimized via an MM2 force field [30].

### 2.3. Structure-Based Virtual Screening (SBVS)

After preparing both the MvfR receptor and the drug libraries, SBVS was performed, targeting the ligand-binding domain of MvfR, which was collectively formed by two subdomains connected through antiparallel β-sheets. The ligand-binding domain was hydrophobic and comprised active residues (Thr265 (X: 21.693 Å, Y: −20.196 Å and Z: 5.844 Å) and Leu189 (X: 15.868 Å, Y: −31.230 Å and Z 0.342 Å)) that were reported to be in regular contact with the M64 co-crystallized compound [16]. Virtual screening of the libraries was achieved using the AutoDock Vina program [31] and GOLD 5.2 [32], where the grid box was centered at the above-mentioned residues with dimensions along the XYZ axes of 25 Å. To be certain about the docking protocol, the co-crystalized ligand was extracted and docked to the MvfR blindly. After confirmation of the docking method, the ligand libraries were screened against the targeted pocket of the MvfR. The number of poses generated for each compound was tuned to 100; these were clustered, and the ones with the lowest binding energy scores and the greatest numbers of hydrophobic and hydrophilic interactions were selected for complex formation. In total, 3 complexes were selected, including one control (M64), for further analysis.

### 2.4. Dynamics Understanding Using Molecular Dynamics Simulations 

**Figure 1:**
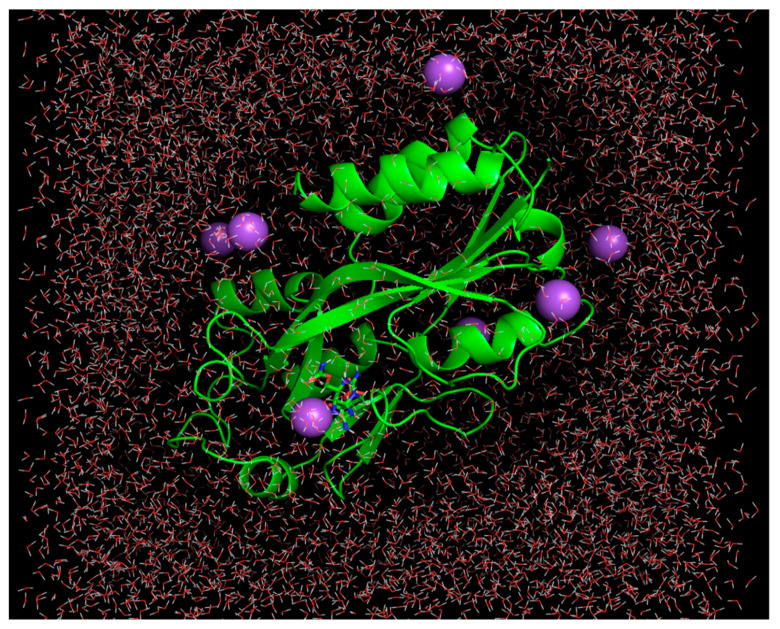
Waterbox containing MvfR-Top-1 lead complex. The MvfR structure is shown via the green cartoon, while Top-1 lead is represented by the green stick. The purple balls are Na^+^ ions while the small red-white sticks are water molecules.

Molecular dynamics simulations were performed to evaluate the binding mode of the leads and the control. Docking results are usually not satisfactory and post-molecular-docking analyses, including molecular dynamics simulation and binding free energies, are widely applied to validate docking predictions [33,34,35,36]. The AMBER20 simulation package [37] was used to perform all atom simulations. The Antechamber program [38] was employed to recognize the atom type and bond type, to find the missing force field parameters and provide similar substitutes, and to generate the topology files. This module was considered to automatically generate drug molecules and protein input parameters for simulation. Further, parametrization of the compounds and the MvfR was conducted using AMBER GAFF [39] and the ff14SB force field [23], respectively. After preparation, each complex was placed in a TIP3P water box of 12 Å dimensions (to ensure that the box size was sufficient to allow proper complex dynamics and that the opposite parts of the complex from the adjacent cells did not see each other), which was then treated with a suitable number of Na^+^ counter ions (9 in total) to obtain a neutral charge system (Figure 1). To prepare the systems for the production run, the complexes were subjected to different phases of energy minimization, as follows: energy minimization for a total of 3000 steps (hydrogen atom energy minimization, water box energy minimization, complex energy minimization, and non-heavy atom minimization) with different set restraints as used previously [40]. The number of cycles of each energy minimization step was adjusted according to the studied systems. Further, an NVT ensemble was used to gradually heat the systems from 0 to 300 K at a time interval of 2 femtoseconds for 20 ps, keeping a constraint of 5 kcal/mol. During this phase, the temperature was kept constant through the use of Langevin dynamics [41]. The SHAKE algorithm [42] was used to constrain the geometry of bonds that involved hydrogen atoms. The systems were then equilibrated using a two-femtosecond time step. After this, each system was treated at constant pressure and temperature for 1 ns, at 1 bar pressure and 300K temperature. Another round of equilibration was performed for 1 ns. The production run was performed for 200 ns. The CPPTRAJ module [43] was applied to perform an analysis of the simulation trajectories in order to evaluate the stability of the system structures. 

### 2.5. Analysis of Radial Distribution Function 

The radial distribution function (RDF) is denoted by *g(r)* and was applied after molecular dynamics simulation to illustrate the variation in the density of interatomic interactions with respect to time [44]. RDF was performed through the CPPTRAJ module of AMBER considering only hydrogen bonds between the compounds and MvfR residues. The hydrogen bond interactions were examined using an in-house Visual Molecular Dynamics (VMD) Perl script [45].

### 2.6. Binding Free Energies Calculation

Complexes were then subjected to the AMBER MMPBSA.py method to calculate the net binding free energies of the complex, enzymes and compounds [46]. The net system binding free energy was considered by subtracting the combined binding energy of the enzyme and compound from the complex binding energy. From simulation trajectories, 1000 frames were picked at a regular time interval and investigated for molecular mechanical energies and solvation free energies. The binding free energy estimation was conducted through two methods: MM-PBSA and MM-GBSA [47,48]. Statistically, both methods estimate binding free energy as,

ΔG net binding free energy=ΔG binding free energy of complex − ΔG receptor+ΔG ligand

### 2.7. Normal Mode Analysis for Assessing Binding Entropy 

The AMBER NMODE module was employed to compute the contribution of entropy to the net binding MM-PB/GBASA energy of the complexes [49]. Only 10 frames of the trajectories were analyzed.

### 2.8. WaterSwap Analysis 

Further confirmation on the intermolecular stability of the MvfR-compound complexes was achieved by estimating the absolute binding free energies using WaterSwap from the Sire Package [50,51]. WaterSwap works on the idea of swapping ligand dimensions at the active pocket of the enzyme with water molecules of equal volume and size from the explicit environment. One thousand iterations were completed for each system, which is reported to be enough to obtain converged binding energy values. The absolute binding free energy was calculated using four efficient methods: thermodynamic integration (TI), free energy perturbation (FEP), quadrature-based integration of TI, and Bennett’s acceptance ratio (BAR) method. The stability of complexes was considered to be high if they had net energy values <1 kcal/mol [52].

### 2.9. Pharmacokinetics Studies

The SwissADME [53] and pkCSM [54] online software applications were used to predict the ADMET properties of the lead molecules. 

## 3. Results and Discussion 

### 3.1. Identification of Potential Leads

SBVS screening studies were performed at the active pocket of the MvfR enzyme using two highly reliable docking software packages: AutoDock Vina and GOLD. The results of both virtual screenings were then sorted on the basis of the docking function, and top two leads (in comparison with the M64 control) were selected. Top-10 hits that were virtually screened and had higher binding affinity binders for the targeted MvfR are tabulated in Table 1. The top two hit compounds: (4-((carbamoyloxy)methyl)-10,10-dihydroxy-2,6-diiminiodecahydropyrrolo[1,2-c]purin-9-yl sulfate) and 10,10-dihydroxy-2,6-diiminio-4-(((sulfonatocarbamoyl)oxy)methyl)decahydropyrrolo[1,2-c]purin-9-yl sulfate were consistently observed to have excellent binding; therefore, they were considered for additional computational analysis. Selection of these compounds in complex with MvfR was conducted considering their structural stability in molecular dynamics simulations and MM-GBSA analysis. The 50-ns MD simulation validated the stability of the docked conformation of the lead compound with MvfR, and the structural deviations of the Cα atoms were plotted against time as the RMSD (presented in Section 3.3). Contrary to the control M64, the screened lead–MvfR complexes were stable and followed a somewhat similar RMSD trend within 2 Å. Small numbers of frames from the molecular dynamics simulations were then selected to estimate the MM–GBSA binding free energy for further validation of the binding strength of the lead molecules for MvfR. The estimated net MM–GBSA binding free energies of Top-1, Top-2 and control M64 were −24.15 kcal/mol, −45.47 kcal/mol and −68.89 kcal/mol, respectively.

### 3.2. Leads and Control Binding Conformation and Interactions

**Figure 2:**
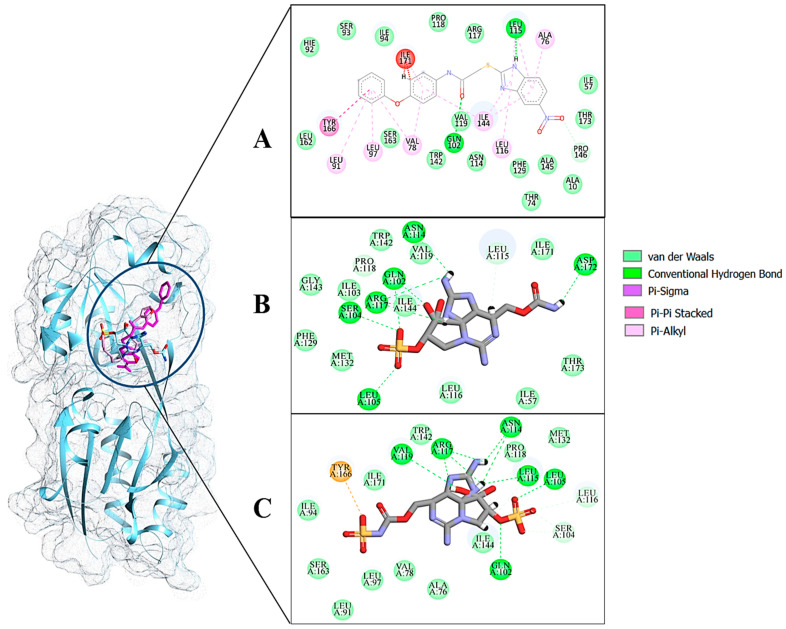
Binding conformation of leads (Top-1 (tan stick) and Top-2 (sky blue stick)) and control (magenta stick) with respect to MvfR (shown in blue cartoon). Residue level interactions of control (**A**), Top-1 (**B**) and Top-2 (**C**).

The AutoDock binding free energy score and GOLD fitness score of the control were −8.14 kcal/mol and 55.14, respectively. The majority of the interactions produced by M64 with MvfR residues were hydrophobic and were similar to those reported previously. In our study, we found that the M64 formed a closed-distance hydrogen bond with Gln102 through the central chemical moiety oxygen atom. This finding is in line with the co-crystallized structure, which is a strong indicator of the soundness of the docking methodology applied herein. The rest of the M64 structure, including Ala10, Ile57, Thr74, Hie92, Ser93, Ile94, Asn114, Arg117, Pro118, Phe129, Trp142, Ala145, Pro146, Leu162, Ser163, and Thr173, was strongly entangled by van der Waals residues. Along with this, several pi–alkyl, pi–sigma and pi–pi stack interactions could be noticed between M64 and MvfR (Figure 2A). M64 is a highly competitive antagonist and has an enhanced affinity for MvfR than natural binding substrates. The Top-1 lead, in contrast to M64, produced a higher number of hydrogen bonds than van der Waals and other chemical contacts. The AutoDock binding energy of the compound was −9.18 kcal/mol and its GOLD fitness value was 61.4. The 10,10-dihydroxy-2,6-diiminiodecahydropyrrolo[1,2-c]purin-9-yl sulfate region, in particular, dominated the bindings and favored the compounds that showed hydrogen bonding with active pocket residues such as Gln102, Ser104, Asn114, and Arg117. The opposite carbamic acid chemical moiety of the compound favored the formation of hydrogen bonds with Asp172. The van der Waals interactions of this compound involved the residues Ile57, Ile103, Leu116, Pro118, Val119, Trp142, Gly143, Ile144, Ile171, and Thr173 (Figure 2B). The Top-2 compound had a GOLD score of 59.2 and a binding energy value of -9.0 kcal/mol. Similarly to Top-1, Top-2 dominated its interactions with the MvfR through hydrogen bonds. The compound formed hydrogen bonds with Gln102, Leu105. Asn114, Leu115, Arg117, and Val119. The rest of the interactions can be seen in Figure 2C. Overall, the docking study predicted that the lead compounds and the control would favor binding to the same ligand-binding domain and produce interaction networks of the same kind.

### 3.3. Deciphering Conformational Dynamics

**Figure 3:**
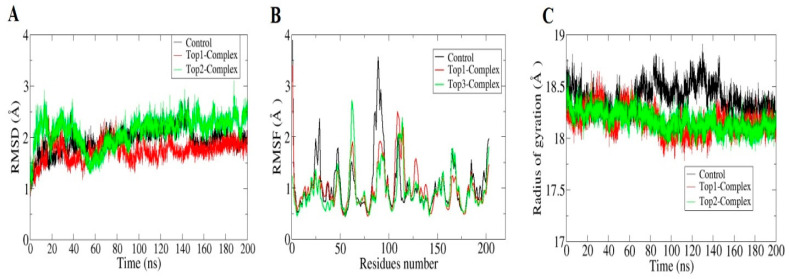
Molecular dynamics simulation trajectories analysis to evaluate the stability of the complexes’ dynamics as a function of time. (**A**) RMSD analysis. (**B**) RMSF analysis sand radius of gyration analysis (**C**) of MvfR in the presence of co-crystallized control, Top1 and Top2 compounds.

The initial 50 ns of the molecular dynamics simulation was extended to 200 ns to obtain confidence values of the complexes’ stability and the strength of leads’ interactions with the MvfR. Different analyses conducted on the simulation trajectories of the leads and control trajectories are presented in Figure 3. Molecular dynamic simulation is a highly useful technique for determining the time-dependent stability of the ligand–receptor interactions and docked conformation. Different statistical analyses based on Cα atoms were performed, such as the radius of gyration (Rg) [55], the root mean square deviation (RMSD) [56] and root mean square fluctuations (RMSF) [57]. Analyses were started by calculating the RMSD for all three complexes by superimposing all frames of the molecular dynamics simulation over the initial docked conformation, and a plot was generated for the entire simulation time using the XMGRACE software [58]. From Figure 3A, consistent stability of the systems could be witnessed and the systems’ RMSD were close to the 2 Å mark throughout the simulation time. This indicates that the leads were enjoying the affinity inside the active pocket of the MvfR, similarly to that of the co-crystalized M64, and were strongly attached to the active side residues via hydrophobic and hydrophilic interactions. The different interactions that allowed stable binding of the compounds at the enzyme’s active pocket are mentioned in Table 2 and the Radial Distribution Function section. For compound 1, Leu71, Tyr73, Arg197, and Leu200 were reported to be in consistent contact with the ligands, while Ser104, Leu115, Arg117, Ser163, Gln190, and Ile194 were reported for compound 2. Due to the stable binding conformation of the compounds during the simulation time, the interacting residue pattern remain mostly the same and did not experiences any major shifts. Next, RMSF analysis was performed on the Cα atoms, visualizations of which are shown in Figure 3B. All the systems’ residues revealed highly stable RMSF values and were within the range of <2 Å with the exception of a few 3 Å spikes. It was observed that the loop regions of MvfR represented high fluctuations in the presence of compounds/control; however, they were still in an acceptable range and allowed proper accommodation of the leads/control inside the pocket for enhanced docked stability. Lastly, the MvfR’s compactness and structure equilibrium were tested in the presence of the leads/control by means of Rg analysis of the Cα atoms. As can be noticed in Figure 3C, all three systems were in good equilibrium (<19 Å). The lead complexes, in particular, were more stable than the control complex. In summary, the simulation analysis confirmed the stability of the systems and identified the compounds as suitable candidates for further experiments in order to unravel their real affinity for the MvfR.

### 3.4. Analysis of the Hydrogen Bonds

**Figure 4:**
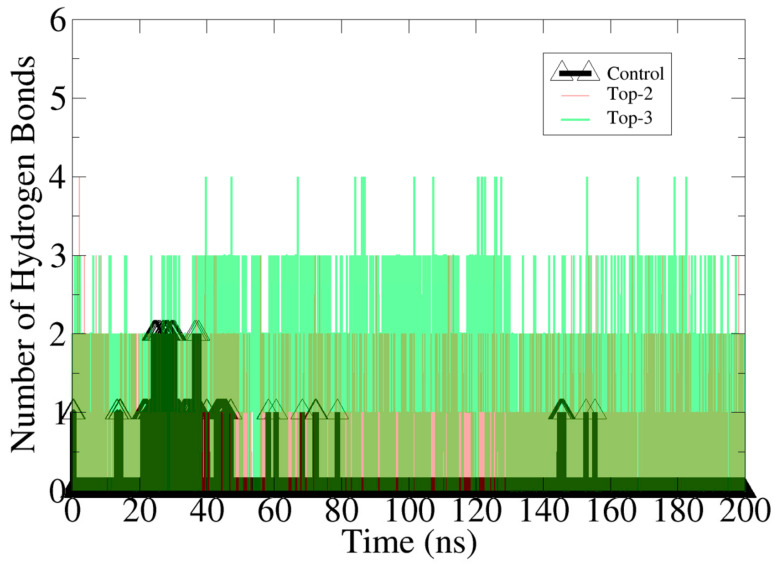
Number of hydrogen bonds produced by compounds with the enzyme during simulation time.

In order to find out the occupancy of hydrogen bonding between the ligand molecules and the protein, we performed hydrogen bond analysis [59]. These interactions determined the intermolecular specificity and were important to stabilize the ligand–protein complexes. The formation of hydrogen bonds for control, Top-1 and Top-2 was plotted using a cut-off of 3.0 Å and a 20-degree cut-off angle in Visual Molecular Dynamics v.1.93 (VMD). The numbers of hydrogen bonds formed by control, Top-1 and Top-1 with the enzyme during the simulation time are shown in Figure 4. The occupancy of different hydrogen bonds formed by Top-1 and Top-2 leads with MvfR are listed in Table 2. The control, Top-1 and Top-2 leads were found to form 12, 68, and 28 hydrogen bonds, respectively, with the MvfR. Several key residues already predicted by molecular docking studies were unveiled to play crucial roles in ligand binding throughout the length of the simulation time. Several previous studies reported the importance of hydrogen bonds while designing new drug molecules against a given biological target [60,61]. For example, Khalid et al. [24] demonstrated several key residues of soluble guanylate cyclase H-NOX domain with ligand molecules.

### 3.5. Radial Distribution Function (RDF) Analysis

**Figure 5:**
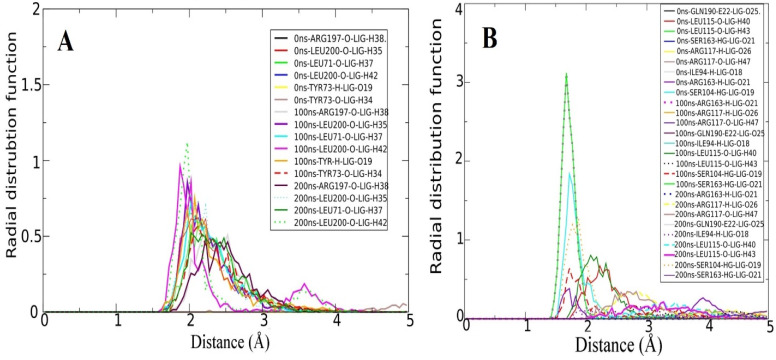
RDF plots of interactions between MvfR and leads that were constantly noticed during molecular dynamics simulation. (**A**) Top-1 lead. (**B**) Top-2 lead.

Furthermore, the RDF analysis was conducted using strong intermolecular interactions between MvfR and the compounds to understand the intensity of interactions versus time. RDF has been frequently employed in studies to highlight the critical intermolecular interactions that are key in the recognition and binding of good affinity binders [9,57,62]. Several residues were filtered that favored continuous contacts with the compounds throughout the simulation time (Figure 5). These interactions were plotted in terms of density versus distance. In the case of Top-1, residues such as Leu71, Tyr73, Arg197, and Leu200 were among the high-density interactions with MvfR, while, in the case of Top-2, Ser104, Leu115, Arg117, Ser163, Gln190, and Ile194 were among the high-density residues that were in consistent interactions. Interactions that remained constant after specific time periods are not provided while those of bond distance variations are plotted.

### 3.6. Assessment of MM-GB/PBSA Binding Free Energies

The estimation of binding free energy via the MM-PBSA and MM-GBSA gives reliable predictions about a compound’s affinity for a given biological macromolecule [47,48]. Both of the mentioned techniques are widely employed in drug design processes because of their low computational demands and the fact that the results can be easily correlated to experimental results [63]. Both of the methods have been regularly employed to validate molecular dynamics simulations and docking predictions [64,65,66]. Complete results of the binding free energies of the complexes are tabulated in Table 3. For both the lead and control complexes, electrostatic and van der Waals energy were revealed to play a critical role in binding, as predicted by the docking findings. The control was found to show higher van der Waals domination in net interactions compared to the lead molecules, which exhibited higher electrostatic contributions as well equal contributions from van der Waals energy. The solvation energy in the case of the control was found to be more dominated by non-polar energy, whereas, for the leads, the polar solvation energy was three times more stabilized than the non-polar solvation energy. Overall, the net binding energies of the systems were very high, thus demonstrating the stability of the systems. The net binding free energies of the systems are in following order: control (MM–GBSA (−41.7 kcal/mol), MM–PBSA (−31.6 kcal/mol)), Top-1 (MM–GBSA (−76.3 kcal/mol), MM–PBSA (−80.8 kcal/mol)), and Top-2 (MM–GBSA (−143.8 kcal/mol), MM–PBSA (−149.1 kcal/mol)). For Top-1, electrostatic energy was found to be predominant in the docking studies as the hydrogen bond distances were very close, mostly within 3 Å. The hydrophobic interactions were also reported to equally favor the stable binding of compounds and played a key role, along with hydrophilic interactions, in holding the compound conformation at the active pocket. Both MM/GBSA and MM/PBSA agree on the significant electrostatic contribution and equally favorable binding of the van der Waals energy. In dynamics simulation analysis, the same findings were revealed, where both the hydrophilic and hydrophobic interactions (as shown in Table 2 and RDF analysis) remained critical for the stability of the RMSD plot. In case of Top-2, the van der Waals energy dominated over the electrostatic energy by a very low margin; the same was observed in the docking analysis. The van der Waals and other hydrophobic interactions pushed the more electronegative chemical moieties of the compound towards the inside of the pocket. This resulted in good interaction networks of both the electrostatic and van der Waal contacts. The binding conformation stabilities and binding interaction profiles of the compounds with the enzyme remained consistent in all of the analyses performed in this study, all of which classified the compounds as strong binders of MvfR.

### 3.7. MvfR Hotspot Residues

Further analysis was conducted to determine the key hotspot residues of MvfR that contributed significantly in terms of binding and holding the leads/control at the active pocket. Identification of hotspot residues was performed in many previous studies to report key interactions between ligands and residues that were vital in stabilizing the ligands at the docked site [57,67]. The net MM-GBSA binding energies of the systems were decomposed into residues of the MvfR, and only the common residues that were critical in binding the ligands were shortlisted, as shown in Table 4. Gln102, Asn114, Arg117 and Val199 were common in all complexes and were found to be major contributors to the ligand interactions. Gln102 was a key hydrogen bonding residue and was reported previously in hydrogen-bonding interactions with ligand leads. It was observed that the rest of the residues involved both hydrogen bonding as well as van der Waals interactions.

### 3.8. Calculating Binding Entropy

To compensate for the missing approximation of binding entropy in MM-PBSA and MM-GBSA, the entropy calculation was implemented via normal mode in the AMBER package. As the calculation was very slow, only a limited number of frames were analyzed. The net entropy of the systems was in the following order: control (−8.89 kcal/mol), Top-1 (−10.10 kcal/mol) and Top-2 (−11.00 kcal/mol).

### 3.9. Evaluation of WaterSwap Absolute Binding Free Energy

**Figure 6:**
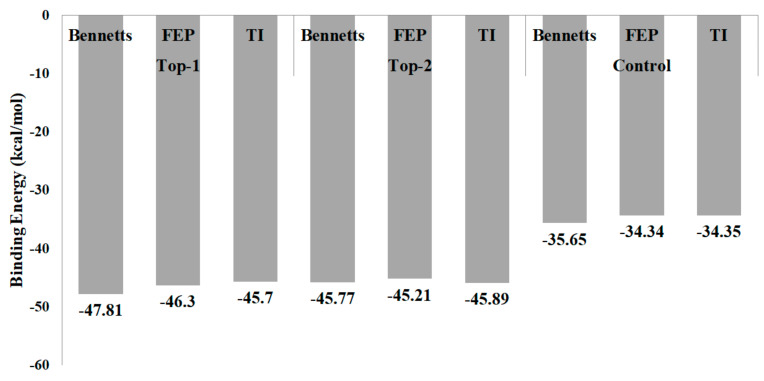
Binding energy values (kcal/mol) calculated by different methods in WaterSwap.

Although the MM-PBSA and MM-GBSA methods are very successful in determining free energies, they have several limitations; therefore, another validation method, WaterSwap, was applied in the study. The WaterSwap-based binding free energy values, calculated using different algorithms, are illustrated in Figure 6. Both of the lead molecules were disclosed as better binders than control M64. As can be seen, the net WaterSwap energies calculated the using algorithms for all three systems differed by no more than 1 kcal/mol, which demonstrated highly converged systems.

### 3.10. Leads Pharmacokinetics

Unfavorable pharmacokinetics of compounds in the process of drug discovery can lead to drug failure, and thus, can increase the time and cost involved in the development of potent and safe drugs [53,54]. For this purpose, pharmacokinetics predictions are important in the early stages of drug development using available in silico tools to enhance the chances of selecting the correct molecules for development. Medicinal chemistry focuses on drug absorption, and this was evaluated as the first step in these in silico studies. It was observed that both compounds were highly water soluble, as predicted by the ESOL, Ali and SILICOS-IT methods in the SWISSADME server. For this reason, the compounds are excellent candidates in terms of oral bioavailability. Further, the compounds had no Pan-assay interference compounds (PAINS) structure; thus, they targeted one specific biological target and had one desired effect [68]. From a synthetic chemistry perspective, the compounds had a good synthetic accessibility score of ~5, meaning they will be easy to synthesize in future experimental analyses. The compounds also had high gastrointestinal absorption and did not act as substrates for the P-glycoprotein transporters. The transdermal deliveries of the compounds are also predicted to be very good, making them suitable for skin-related products. They had volume distribution values that indicated their low tissue distribution as compared to their distribution in the plasma. Likewise, they also had low fraction unbound values, which indicate that they could lower their serum protein binding affinities and could enhance their distribution efficiency through the cell membranes. The blood–brain barrier crossing abilities of drugs are important in terms of evaluating their side effects and toxicity, as well as the efficiency of their pharmacological action in the brain [69]. These compounds had poor blood–brain barrier penetration, and thus, they could not move through the central nervous system easily. Additionally, they did not inhibit the detoxification of cytochrome P450, and thus, were involved in the oxidation of xenobiotics to help in their removal. The renal and hepatic clearance of the compounds were projected to be ~0.53 log mL/min/kg. This total clearance of compounds is an important factor in terms of evaluating their bioavailability and calculating the rate of dosage for their steady-state concentration. They were found to be AMES non-toxic based on their LD50 values during oral administration to rats, and were anticipated to demonstrate no sensitization of the skin and to not inhibit hERGI and hERGII, which can reduce the likelihood of QT syndrome development. Detailed pharmacokinetic data of both lead molecules are tabulated in Table 5.

## 4. Conclusions

Computer aided drug design is an integral part of modern drug discovery and has played a significant role in the development of drugs that are in clinical trials and in clinical use [17]. In this study, we identified two leads against MvfR of *P. aeruginosa*, which, as with the co-crystallized M64, had high binding affinity for the relevant enzyme. Additionally, the compounds met prominent druglike rules and had good pharmacokinetics and acceptable safety. The docking and subsequent molecular dynamics simulations revealed the formation of strong interactions by the compounds with active pocket residues and significant conformation stability. There were multiple van der Waal and hydrogen bond interactions of the compounds with the hotspot residues of the pocket, which resulted in increased intermolecular affinity. In particular, strong van der Waals interactions and hydrogen bonding was observed between all the screened molecules and MvfR residues (Gln102, Asn114, Arg117, Val119 and Asp172). These residues were present in all the crystal structures of the MvfR in P. aeruginosa and formed bonds with co-crystallized ligands [16]. These residues are conserved among the different MvfR and are considered to be key for enzyme functionality [16]. Thus, there are fewer chances for the enzyme to escape the compound’s action. In addition, the compounds revealed favorable druglike and lead-like properties and were reported to have good pharmacokinetic profiles. This also increases the chances of the compounds reaching the market. Since these compounds showed promising results and are easily available from commercial sources, they can be used in further quick in vivo experiments to determine their real binding affinity and MvfR inhibition potential. Moreover, it is suggested that the anti- *P. aeruginosa* activity of the compounds be investigated to affirm that the molecules do not infer with the essential biological pathways of the pathogens and do not harm bacterial cells. This will ensure that the compounds will only de-weaponize the pathogen and will not mediate the evolution of new resistant mechanisms. To summarize, these screened compounds provide promising starting structures in the search for novel anti-virulent compounds against the superbug *P. aeruginosa*. Although very stringent criteria for docking score functions, as well as for the dynamics simulations and binding free energy methods, were applied for the compounds in this study, the shortlisting and selection of stable conformations can still be undertaken with confidence, and the selection of the ligand conformation can be enhanced via such approaches as using virtual screening rather than docking-based screening in long-length traditional molecular dynamics simulations [70,71], thoroughly analyzing the binding of free energies throughout the frames of simulations, using the hybrid QM/MM approach for further enhancing the prediction of stable conformation accuracy [72], using the Selective Ligand-Induced Conformational Ensemble (SLICS) method [73], using different analytical methods such as axial frequency distribution (AFD) [74] and the Binding Free Energy-Based Footprint Pharmacophore Model method [75], etc.

## Figures and Tables

**Table 1:** GOLD fitness score and binding free energy of compounds for the MvfR enzyme.

#	Docked Complexes	Gold Score	AutoDock Binding Energy Value
1	Top-1 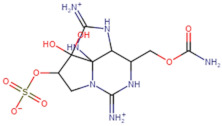	61.4	−9.18
2	Top-2 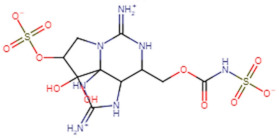	59.2	−9.0
3	Top-3 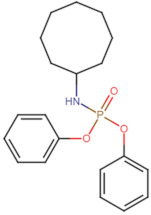	58.4	−9.14
4	Top-4 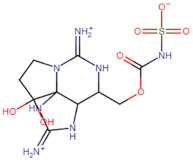	57.2	−8.4
5	Top-5 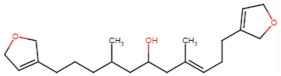	56.3	−9.10
6	Top-6 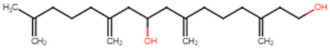	56.2	−7.88
7	Top-7 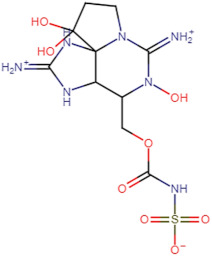	55.3	−7.37
8	Top-8 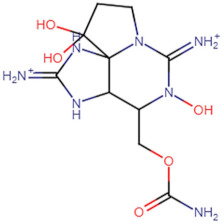	54.4	−7.19
9	Top-9 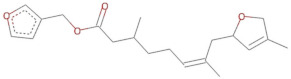	54.3	−7.01
10	Top-10 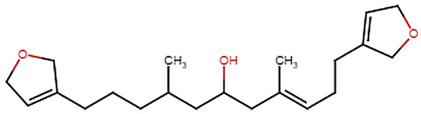	52.2	−6.78

**Table 2:** Occupancy of hydrogen bonds formed between the leads and the MvfR enzyme.

Donor	Acceptor	Occupancy (%)
**Control**		
LIG204-Side-N3	LEU115-Main-O	0.13%
TYR166-Side-OH	LIG204-Side-O3	0.19%
LIG204-Side-N3	ASP172-Main-O	4.06%
THR173-Side-OG1	LIG204-Side-N4	0.98%
LYS174-Side-NZ	LIG204-Side-O3	0.01%
TYR73-Side-OH	LIG204-Side-C8	0.01%
TYR166-Side-OH	LIG204-Side-C6	0.01%
LIG204-Side-N3	TYR166-Side-OH	0.02%
LIG204-Side-N3	GLU167-Side-OE1	0.09%
LIG204-Side-N3	GLU167-Side-OE2	0.01%
LIG204-Side-N2	ARG117-Main-O	0.15%
LIG204-Side-N2	LEU115-Main-O	0.01%
**Top-1**		
TYR166-Side-OH	LIG204-Side-O18	0.48%
LIG204-Side-N17	THR173-Side-OG1	0.82%
LYS174-Main-N	LIG204-Side-O19	1.81%
LYS174-Side-NZ	LIG204-Side-O19	0.33%
LIG204-Side-N16	ASN114-Main-O	0.13%
LIG204-Side-N12	ASP172-Main-O	0.15%
TYR166-Side-OH	LIG204-Side-O22	0.23%
LIG204-Side-N12	THR173-Side-OG1	0.01%
TYR166-Side-OH	LIG204-Side-O23	0.02%
TYR166-Side-OH	LIG204-Side-O21	0.06%
LIG204-Side-N17	ASP172-Main-O	0.14%
TYR166-Side-OH	LIG204-Side-O25	0.01%
LIG204-Side-N12	ASP172-Side-OD1	2.38%
LIG204-Side-N15	ASP172-Side-OD2	3.11%
LIG204-Side-N17	ASP172-Side-OD1	0.10%
TYR73-Side-OH	LIG204-Side-N11	0.01%
LIG204-Side-N11	TYR73-Side-OH	0.06%
TYR73-Main-N	LIG204-Side-O19	3.09%
LIG204-Side-N17	LEU200-Main-O	4.27%
LIG204-Side-N17	LEU203-Side-OXT	3.56%
LIG204-Side-N11	TYR73-Main-O	2.12%
ARG201-Side-NH2	LIG204-Side-O20	0.09%
LIG204-Side-N12	LEU200-Main-O	0.76%
LIG204-Side-N15	LEU71-Main-O	2.22%
ARG201-Side-NH1	LIG204-Side-O20	0.01%
ARG201-Side-NH1	LIG204-Side-O25	0.14%
LIG204-Side-N15	ARG197-Main-O	0.34%
LIG204-Side-N17	LEU203-Main-O	1.29%
LIG204-Side-N17	ARG201-Main-O	0.05%
LIG204-Side-N16	THR74-Side-OG1	0.03%
ARG201-Side-NH2	LIG204-Side-O18	0.06%
ARG201-Side-NH2	LIG204-Side-O21	0.06%
ARG201-Side-NH1	LIG204-Side-O21	0.02%
ARG201-Side-NH1	LIG204-Side-O22	0.05%
LIG204-Side-N15	THR74-Main-O	0.01%
ARG201-Side-NH1	LIG204-Side-O18	0.04%
ARG201-Side-NH1	LIG204-Side-O23	0.03%
LIG204-Side-N11	TYR73-Side-CD2	0.01%
ARG201-Side-NH2	LIG204-Side-O22	0.01%
ARG201-Side-NE	LIG204-Side-O18	0.01%
TYR73-Side-OH	LIG204-Side-O19	0.05%
LIG204-Side-N15	ASP172-Side-OD1	0.97%
LIG204-Side-N12	ASP172-Side-OD2	0.02%
LIG204-Side-N17	ASP172-Side-OD2	0.01%
LIG204-Side-N15	TYR73-Side-CG	0.01%
LIG204-Side-O23	ASP172-Side-OD1	0.71%
LIG204-Side-O23	ASP172-Side-OD2	0.25%
LYS75-Side-NZ	LIG204-Side-O23	4.95%
LYS75-Side-NZ	LIG204-Side-C1	7.03%
LIG204-Side-N15	TYR73-Side-OH	0.23%
LYS75-Side-NZ	LIG204-Side-N16	0.61%
LIG204-Side-N16	THR74-Main-O	0.07%
LYS75-Side-NZ	LIG204-Side-N11	0.57%
LYS75-Side-NZ	LIG204-Side-O20	0.22%
LIG204-Side-N12	TYR73-Side-CD2	0.08%
LIG204-Side-N12	TYR73-Side-CG	0.01%
LYS75-Side-NZ	LIG204-Side-N13	0.02%
LIG204-Side-O22	ASP172-Side-OD2	0.15%
TYR73-Side-OH	LIG204-Side-N15	0.01%
LIG204-Side-N12	TYR73-Side-CE2	0.01%
LIG204-Side-O22	ASP172-Side-OD1	0.02%
LYS75-Side-NZ	LIG204-Side-O21	0.07%
LIG204-Side-N11	TYR73-Side-CB	0.01%
LYS75-Side-NZ	LIG204-Side-O18	0.02%
LIG204-Side-N17	TYR73-Side-CD2	0.01%
LIG204-Side-N11	ASP172-Side-OD2	0.01%
THR74-Side-OG1	LIG204-Side-N11	0.01%
ARG201-Side-NE	LIG204-Side-O20	0.02%
**Top-2**		
SER104-Side-OG	LIG204-Side-O19	15.02%
SER163-Side-OG	LIG204-Side-O18	5.49%
LIG204-Side-N13	LEU115-Main-O	1.27%
LIG204-Side-N16	ASN114-Main-O	0.14%
LIG204-Side-O26	LEU115-Main-O	0.01%
SER163-Side-OG	LIG204-Side-O21	42.57%
GLN102-Side-NE2	LIG204-Side-O25	0.07%
SER163-Side-OG	LIG204-Side-O22	2.19%
GLN102-Side-NE2	LIG204-Side-O19	0.29%
SER104-Side-OG	LIG204-Side-O24	10.23%
GLN102-Side-NE2	LIG204-Side-O24	1.74%
TYR166-Side-OH	LIG204-Side-O20	18.22%
SER104-Side-OG	LIG204-Side-O23	4.27%
ILE144-Main-N	LIG204-Side-O19	0.01%
TYR166-Side-OH	LIG204-Side-O22	16.34%
GLN102-Side-NE2	LIG204-Side-O23	1.74%
LIG204-Side-O26	ARG117-Main-O	0.52%
ARG117-Main-N	LIG204-Side-O26	0.14%
LIG204-Side-N15	VAL78-Side-CG2	0.02%
TYR166-Side-OH	LIG204-Side-O21	6.73%
TYR166-Side-OH	LIG204-Side-O18	5.83%
ILE94-Main-N	LIG204-Side-O18	1.08%
ILE94-Main-N	LIG204-Side-O22	1.31%
ILE94-Main-N	LIG204-Side-O21	0.65%
SER93-Side-OG	LIG204-Side-O22	0.03%
SER93-Side-OG	LIG204-Side-O21	0.04%
LIG204-Side-N17	ASP172-Main-O	0.04%
LIG204-Side-N17	THR173-Side-OG1	0.03%

**Table 3:** Estimated net binding energies (in kcal/mol) of complexes at different time steps of molecular dynamics simulation trajectories.

Compound	MM/GBSA						
	ΔG Binding	ΔG Electrostatic	ΔG Bind Van Der Waals	ΔG Bind Gas Phase	ΔG Polar Solvation	ΔG Non-Polar Solvation	ΔG Solvation
Control	−41.7	−6.9	−54.6	−61.6	26.5	−6.6	19.9
Top-1	−76.3	−30.6	−25.1	−55.7	−17.4	−3.2	−20.6
Top-2	−143.8	−23.4	−39.9	−63.3	−75.0	−5.5	−80.5
	**MM/PBSA**						
Control	−31.6	−6.9	−54.6	−61.6	34.6	−4.6	30.0
Top-1	−80.8	−30.6	−25.1	−55.7	−22.5	−2.6	−25.1
Top-2	−149.1	−23.4	−39.9	−63.3	−81.9	−3.9	−85.8

**Table 4:** Critical hotspot residues that contributed heavily in the interactions with the MvfR residues.

Residue	Control	Top-1	Top-2
Gln102	−2.1	−6.88	−8.14
Asn114	−3.4	−7.01	−6.40
Arg117	−1.8	−5.78	−8.49
Val119	−2.8	−6.41	−9.78
Asp172	−1.74	−2.87	−9.14

**Table 5:** Detailed pharmacokinetic data of lead molecules.

Property	Model Name	Predicted Value Top-1	Predicted Value Top-2	Unit
Absorption	Water solubility	−2.892	−2.892	Numeric (log mol/L)
Absorption	Caco2 permeability	−0.601	−0.673	Numeric (log Papp in 10^−6^ cm/s)
Absorption	Intestinal absorption (human)	0	0	Numeric (% Absorbed)
Absorption	Skin Permeability	−2.735	−2.735	Numeric (log Kp)
Absorption	P-glycoprotein substrate	Yes	Yes	Categorical (Yes/No)
Absorption	P-glycoprotein I inhibitor	No	No	Categorical (Yes/No)
Absorption	P-glycoprotein II inhibitor	No	No	Categorical (Yes/No)
Distribution	VDss (human)	0.01	−0.005	Numeric (log L/kg)
Distribution	Fraction unbound (human)	0.382	0.387	Numeric (Fu)
Distribution	BBB permeability	−2.587	−2.775	Numeric (log BB)
Distribution	CNS permeability	−7.316	−6.425	Numeric (log PS)
Metabolism	CYP2D6 substrate	No	No	Categorical (Yes/No)
Metabolism	CYP3A4 substrate	No	No	Categorical (Yes/No)
Metabolism	CYP1A2 inhibitor	No	No	Categorical (Yes/No)
Metabolism	CYP2C19 inhibitor	No	No	Categorical (Yes/No)
Metabolism	CYP2C9 inhibitor	No	No	Categorical (Yes/No)
Metabolism	CYP2D6 inhibitor	No	No	Categorical (Yes/No)
Metabolism	CYP3A4 inhibitor	No	No	Categorical (Yes/No)
Excretion	Total Clearance	0.348	0.34	Numeric (log ml/min/kg)
Excretion	Renal OCT2 substrate	No	No	Categorical (Yes/No)
Toxicity	AMES toxicity	No	No	Categorical (Yes/No)
Toxicity	Max. tolerated dose (human)	0.439	0.438	Numeric (log mg/kg/day)
Toxicity	hERG I inhibitor	No	No	Categorical (Yes/No)
Toxicity	hERG II inhibitor	No	No	Categorical (Yes/No)
Toxicity	Oral rat acute toxicity (LD50)	2.482	2.482	Numeric (mol/kg)
Toxicity	Oral rat chronic toxicity (LOAEL)	6.023	4.58	Numeric (log mg/kg_bw/day)
Toxicity	Hepatotoxicity	No	No	Categorical (Yes/No)
Toxicity	Skin sensitization	No	No	Categorical (Yes/No)
Toxicity	T. Pyriformis toxicity	0.285	0.285	Numeric (log ug/L)
Toxicity	Minnow toxicity	7.668	8.464	Numeric (log mM)

## Data Availability

The data presented in this study are available within the article.
